# Bacterioruberin (C_50_ Carotenoid): Nutritional and Biomedical Potential of a Microbial Pigment

**DOI:** 10.3390/nu17243899

**Published:** 2025-12-12

**Authors:** Rosa María Martínez-Espinosa

**Affiliations:** 1 Multidisciplinary Institute for Environmental Studies “Ramón Margalef”, University of Alicante, Ap. 99, 03080 Alicante, Spain; rosa.martinez@ua.es; Tel.: +34-965-903400 (ext. 1258 or 8841); 2 Biochemistry and Molecular Biology and Edaphology and Agricultural Chemistry Department, Faculty of Sciences, University of Alicante, Ap. 99, 03080 Alicante, Spain

**Keywords:** C_50_ carotenoids, bacterioruberin, haloarchaea, antioxidant, immunomodulation, antitumoral, skin protection, anti-atrophy effect, antilipidic, antiglycemic, nutraceuticals

## Abstract

Haloarchaea are moderate and extreme halophilic microorganisms inhabiting hypersaline environments characterised by high ionic and oxidative stress due to extremely high salt concentrations and high incidence of UV radiation (mainly in spring and summer). To be alive and metabolically active under these harsh conditions, haloarchaeal strains have developed molecular adaptations, like hyperpigmentation. Among the carotenoids produced by haloarchaeal species, the C_50_ carotenoid called bacterioruberin (BR) and its derivatives, monoanhydrobacterioruberin and bisanhydrobacterioruberin, are the predominant natural pigments produced. This review aims to highlight the most significant characteristics of BR and their derivatives, as well as a description of the biological activities already reported that could provide benefits for human health, including antitumoral, immunomodulatory, antioxidant, skin protectant, antilipidemic, antiglycemic, and anti-atrophic effects, in addition to showing potential positive effects on sperm cells cryopreservation. Overall, C_50_ carotenoids are fascinating natural biomolecules that could be utilised in processed food and nutraceuticals or as tools in the context of new strategies and/or pharmaceutical formulations to combat various human diseases or metabolic disorders.

## 1. Introduction

Extremophile environments are important for understanding the limits of life, with applications in biotechnology, and for informing astrobiology and space exploration. There are different types of extremophilic ecosystems, such as hypersaline ecosystems, which are spread worldwide. Hypersaline environments include ecosystems showing salt concentrations higher than those of seawater (3.5% *w*/*v* in seawater vs. up to 35% *w*/*v* in brines). Some of the most representative hypersaline ecosystems that occupy vast geographical extensions include salterns, inland lakes, lagoons, marshes, and coastal/inland salty ponds (also termed salt flats or salt pans) [1]. In brines or soils of these ecosystems characterised by the presence of very high salt concentrations (between 2 and 4 M NaCl or equivalents), moderate or extreme halophilic microorganisms of the archaea domain (haloarchaea, families *Halobacteriaceae* and *Haloferacaceae*) constitute the predominant microbial communities, all together with the bacteria of the genus *Salinibacter* (which is the most abundant) and the unicellular green algal genus *Dunaliella* [2,3].

As a general feature, extreme halophilic microorganisms have evolved to adapt and optimise their molecular machineries to the different biotic and abiotic factors characterising hypersaline ecosystems, most of which cause cellular and molecular stresses in most living beings (high UV radiation, high ionic strength, low water and oxygen availability, nutrient scarcity, etc.) [1,4,5,6,7,8]. To be isosmotic with their surroundings, these microorganisms can follow one of the following two strategies: the “salt-in” strategy, where they accumulate high concentrations of intracellular potassium ions to match external salinity, and the “osmolyte” strategy, where they accumulate organic compounds like ectoine and glycine betaine to balance the osmotic pressure [1,5,6]. Because of these molecular and physiological adaptations, most biomolecules of halophilic microorganisms exhibit unique biochemical parameters and behaviours that distinguish them from their non-halophilic counterparts; examples include the following: (i) higher content of acidic amino acids in protein surface (aspartate, glutamate) and short, polar amino acids that enhance solubility in high salt environments. They have a lower proportion of bulky, hydrophobic amino acids; (ii) a higher number of G-C pairs in DNA molecules, which increases their thermal and chemical stability; and (iii) specialised ion pumps for salt regulation [1,2,3,4,5,6].

Consequently, thanks to these molecular differences, biomolecules from halophilic microorganisms, like enzymes, proteins, exopolysaccharides, lipids, and carotenoids, are promising targets from a biotechnological perspective because they offer some advantages or benefits compared to their non-halophilic homologues [1,9]. In this context, many studies recently reported that using haloarchaea as model microorganisms for biotechnology has confirmed that they can be easily used as cell factories to upscale processes aiming at the production of marketed biomolecules like biopolymers (mainly polyhydroxyalkanoates), enzymes (that show the highest catalytic activity at high ionic strength and high temperatures), antimicrobials (most of them small peptides, like halocins), carotenoids (natural pigments), compatible solutes (like ectoine), lipids, antiadhesives, and biofuels [1,10]. Related to potential applications in pharma industries and in biomedicines of biomolecules synthesised by haloarchaea, C_50_ carotenoids called bacterioruberin (BR), bisanhydrobacterioruberin (BABR), and monoanhydrobacterioruberin (MABR) stand out for their biological activities, from which positive effects on human health have been recently reported [11].

This review aims to highlight the most relevant characteristics of the natural carotenoids BR, BABR, and MABR (although the last two have been described to a lesser extent), as well as a description of the biological activities already reported that could provide benefits for human health: antitumoral, immunomodulatory, antioxidant, skin protectant, antilipidemic, antiglycemic, and anti-atrophic, apart from showing potential positive effects on sperm cells. Most of the works described here correspond to those reported by using haloarchaea as a natural source of BR, BABR, and MABR, but research conducted with BR from bacterial strains has also been integrated in this review. Overall, these carotenoids are promising natural biomolecules with potential applications in the design and development of new approaches and/or pharmaceutical formulations to combat various human diseases or metabolic disorders.

## 2. Materials and Methods

### Search Strategy and Information Processing

This review was conducted as follows, using the following databases: PubMed and Scopus (original articles, reviews, systematic reviews, and meta-analyses). The keywords used alone and in combination were bacterioruberin, archaea, bacteria, and biotechnology. The PubMed advanced form of search was used by selecting the options “title/abstract”, specific date, and languages; in “Scopus”, the option of “document search”, “article title, abstract, keywords” and specific date was used; in “Web of Science”, the basic search was conducted, and the following options were selected: theme, specific date, and “article”. Other aspects, like date or language of the publications, were not specified, and the search was without restriction of date of publication to compile as many documents as possible (except proceedings, which were the only exclusion criteria used).

## 3. Description of Bacterioruberin and Closely Related Compounds

**Figure 1:**
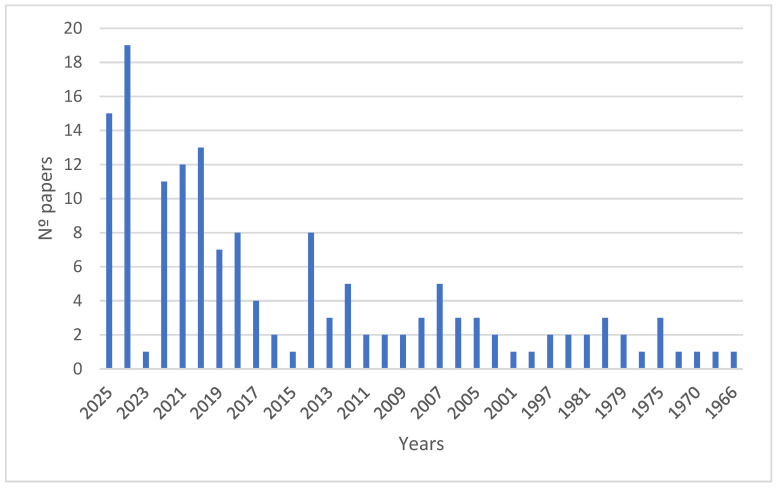
Evolution of the number of indexed international publications on bacterioruberin (according to PubMed; Keyword: bacterioruberin; https://pubmed.ncbi.nlm.nih.gov/?term=bacterioruberin&sort=date, accessed on 28 October 2025).

The first scientific publication on the natural carotenoid BR appeared in the mid-1960s, a time when techniques like NRM and mass spectrometry were not yet available. After this milestone, decades followed in which work was performed on optimising the isolation and characterisation of carotenoid extracts from various haloarchaea and a few bacteria; however, most studies on this carotenoid have been conducted in the last five years, mainly in connection with the description of its biological activities and potential applications in biotechnology (Figure 1).

It was first described as a rare 50-carbon carotenoid that had been isolated from the haloarchaeon *Halobacterium salinarum,* constituting up to 85% of the carotenoid-rich extract isolated at that time. Initially, it was described as a bacterial pigment because there was some controversy as to whether that species was an archaeon or a bacterium [12,13]. Although some studies confirmed that a few extremophilic bacterial species, like the psychrotrophic bacterium *Micrococcus roseus* and *Arthrobacter* species, are also BR producers*,* it is globally accepted that this C_50_ carotenoid and its derivatives are produced almost exclusively by haloarchaeal strains [14,15,16]. Thus, BR is the major carotenoid produced by haloarchaea, followed by monoanhydrobacterioruberin (MABR) and bisanhydrobacterioruberin (BABR) (the last two are dehydrated forms of BR, and therefore, anhydro derivatives of BR, in summary) [17,18].

BR, MABR, and BABR are pigments embedded in the cytoplasmic membranes and usually are isolated by using organic solvents like acetone, methanol, or a mixture of the two solvents [17,18,19,20]. In theory, all three are responsible for several biological activities that are relevant for extreme/moderate haloarchaea to be adapted to life in extreme conditions (as per its location within the cytoplasmic membranes, it protects the cells against UV radiation, low oxygen tension, low water availability, and high ionic strength). However, a large part of the studies published to date attribute biological activities to BR, since this carotenoid is the most abundant in carotenoid-rich extracts isolated from various species of haloarchaea (up to 80–85% of the total as identified by MS-HPLC complemented with UV-VIS spectra). The biological activities reported are of high interest for potential applications in biotechnology and biomedicine [19,20]. Although BR is the major carotenoid synthesised by haloarchaea, other natural pigments have been identified, but at very low concentrations, in haloarchaeal carotenoid extracts (including β-carotene, lycopene, and some xanthophylls, which, in fact, are precursors of BR synthesis according to the carotenogenesis pathway described in haloarchaea) [19,20].

From a chemical point of view, BR is a carotenoid of the xanthophyll group (oxygen-containing carotenoids) consisting of a primary conjugated isoprenoid chain of 50 C atoms that contains 13 conjugated double bonds and four hydroxyl groups arising from the terminal ends. MABR and BABR also consist of a primary conjugated isoprenoid chain of 50 C atoms that contains 13 conjugated double bonds, but the number of hydroxyl groups varies. BR acts as a powerful antioxidant molecule, thus protecting the microbial cells against oxidative stress, thanks to the electron transport flux between the pairs of conjugated double bonds of its chemical structure. Table 1 displays the chemical structure, chemical formula, and complete name of BR, BABR, and MABR [18,19,21,22].

Since BR, MABR, and BABR present a longer hydrocarbon chain and a higher number of conjugated double bonds than other major carotenoids in nature, such as β-carotene (C_40_ carotenoid, nine conjugated double bonds), they have an extraordinary scavenging activity, which is essentially the biological activity that makes them of interest as an antioxidant in several industrial and biomedical sectors [19,20]. Based on the BR chemical composition and structure, it was theoretically assumed that this natural carotenoid shows strong antioxidant properties [19,20]. Most of the other biological activities of BR are molecularly associated with its high antioxidant capacity, which is one of the highest described so far from a natural pigment (it has been estimated that the antioxidant capacity of BR is 300 times higher than the antioxidant activity reported from β-carotene). Apart from the high capacity as a colouring agent and high antioxidant capacity, other recent research publications have demonstrated biological activities of BR with potential applications in biomedicine that are closely dependent on its high antioxidant activity; thus, antitumoral, immunomodulatory, antilipidic, and antiglycemic activities have been proven on human commercial cell lines representative of different pathologies [22,23].

## 4. Potential Effects of Bacterioruberin on Human Health

### 4.1. Antioxidant Properties of Bacterioruberin and Bacterioruberin-Rich Extracts

This is probably the best-characterised biological activity in the case of BR, not only in the context of microbial strains able to synthesise it, but also in in vitro assays aiming at exploring potential effects on commercial cellular lines representative of different human pathologies. In fact, this is the most relevant biological activity, making possible other biological effects like anti-inflammation or apoptosis in tumoral cells (as discussed in Section 4.2 and Section 4.3) [24].

Most of the works focused on the antioxidant activity of BR have been developed with carotenoid extracts isolated from species of the genera *Haloterrigena* [25], *Haloferax* [11,20], *Halobacterium* [26,27,28], *Haloarcula* [29], *Halorhabdus* [30], *Halorubrum*, and *Natronoccoccus* [31,32]. In the case of the *Haloferax* genus, the pigmentation of the cells changed significantly based on the parameters of the incubation and composition of the culture media (pH of the media, low salinity, exposure to light, and/or the presence of compounds like heavy metals, deep eutectic solvents, or prooxidants like H_2_O_2_). From all these works, the main conclusion is that the higher the cellular stress, the higher the pigmentation [11,20,33,34,35]. This observation arose in experiments where growth conditions were optimised to achieve the maximum yield of BR production. Thus, growth conditions in the presence of 2.5% (*w*/*v*) glucose as a carbon source, decreasing optimal salt concentration from 25% to 12.5% (*w*/*v*) salinity, led to a carotenoid-rich extract showing an IC_50_ value of 0.03 μg/mL in the 2,2′Azinobis-(3-Ethylbenzthiazolin-6-Sulfonic Acid assay (ABTS) [23,33,34,35]. On the other hand, it has been reported that the activity of carotenoid extracts from *Haloarcula hispanica* and *Halobacterium salinarum* could scavenge DPPH (2,2-diphenyl-1-picrylhydrazyl) (2.05 μg/mL and 8.9 μg/mL) and ABTS radicals (3.89 μg/mL and low activity for *H. salinarum*) and reduce ferrocyanide and chelate copper. However, processes like NO scavenging or iron chelation were not observed [36]. Works carried out with carotenoid extracts from *Halococcus morrhuae* and *H. salinarum* confirmed values of IC_50_ around 0.85 μg/mL and 0.84 μg/mL for the ABTS assay, respectively [37]. Carotenoid-rich extracts from the haloarchaeon *Halorhabdus utahensis* displayed their scavenging power in a set of different antioxidant assays (DPPH, FRAP, and Superoxide Scavenging Activity assays), confirming that these natural pigments display a wide range of modes of action against oxidants [20,23,30].

Similar methods and approaches (ABTS, FRAP, and DPPH) have been used to quantify the antioxidant activity of *Halorubrum salinarum*, *Natronoccoccus* sp. TC6, and *Halorubrum tebenquichense* carotenoid extracts. The extracts were efficient in all tests, showing relevant capacity for hydrogen and single-electron transfer [38,39,40]. In the case of *Halobacterium salinarum* and *Halobacterium* sp. strain NRC1, BR acts as a protective mechanism against oxidative DNA damage induced not only by UV but also by gamma radiation. Its protective role against desiccation has also been tested in this genus. Therefore, this carotenoid might have potential applications in medicine and cosmeceuticals focused on the mitigation of DNA damage and the preservation of cellular integrity [29,41].

Understanding these protective mechanisms has inspired the design and development of experimental approaches aiming at food preservation or drug/cosmetic formulations for novel therapies, cosmetics, nutraceutics, and skincare products targeting oxidative-stress-induced DNA damage. In connection with the application, recent works have demonstrated that BR extracts can protect skin cells from oxidative DNA-damaging agents, protect erythrocytes from H_2_O_2_, and reduce the concentration of pro-inflammatory cytokines such as TNF-α and IL-6 [42,43]. These findings underscore that BR, and probably other C_50_ carotenoids, have a wide application spectrum, particularly in the mitigation of photoaging [44]. The high antioxidant activity of BR has also been identified as responsible for potential protective effects against muscle atrophy in lipopolysaccharide (LPS)-induced C2C12 myotube atrophy (C2C12 cells correspond to a myoblast cell line that is differentiated into myotubes, i.e., immature muscle fibres, in the lab to simulate muscle tissue) [44]. In C2C12 myotubes, LPS treatment led to a reduction in myotube diameter and number, as well as the hypertranscription of the muscle-specific ubiquitin ligase MAFbx and MuRF1. Under these conditions, the exposure to BR extracts mitigated these changes by activating the Akt/mTOR pathway (also known as the PI3K/Akt/mTOR pathway, a central signalling network that regulates cell growth, proliferation, and survival). Furthermore, BR extracts abolished the elevated cellular reactive oxygen species levels and the inflammation response induced by LPS [44].

Another interesting application of the antioxidant capacity of BR is related to the cryopreservation of sperm cells, which was tested using ram sperm cells. In this research, BR-rich extracts isolated from the haloarchaeon *H. volcanii* strain HVLON3 were assayed on ram sperm after freezing/thawing. To monitor the effect of BR, different physiologically relevant parameters were monitored. Those carotenoid-rich extracts containing between 7 and 20 μmol L^−1^ BR significantly improved the viability of the cells (*p* < 0.0001), total and progressive motility (*p* < 0.0001), and sperm velocities (*p* = 0.0172 for curvilinear velocity VCL, *p* = 0.0268 for average path velocity VAP, and *p* = 0.0181 for straight-line velocity VSL) and did not affect other parameters analysed (like the mitochondrial membrane potential). This applicability is sound in biomedical/biotechnological areas as well as in veterinary fields because it improves the quality of cryopreserved ram sperm cells, thus increasing insemination yields [45].

### 4.2. Antitumoral Properties of Bacterioruberin and Bacterioruberin-Rich Extracts

Given that the antioxidant capacity of BR has been described as one of the most potent for natural carotenoids and starting from the premise that in tumour processes there is a clear imbalance in cellular redox balances, some initial hypotheses pointed out the idea that BR could have some significant effect on the metabolism and proliferation capacity of tumour cells [46,47,48,49,50].

One of the first studies addressing antihaemolytic and antitumoral activity of haloarchaea pigments was published in the middle of the first decade of this century. In this work, the carotenoid extracts from seven halophilic archaeal strains (*Halogeometricum rufum*, *Halogeometricum limi*, *Haladaptatus litoreus*, *Haloplanus vescus*, *Halopelagius inordinatus*, *Halogranum rubrum*, and *Haloferax volcanii*) were characterised from a biochemical point of view, and their biological activities were assayed in mouse erythrocytes (previously stressed by the exposure to H_2_O_2_, which causes haemolysis) and in HepG2 cells (a human liver cancer cell line used in biomedical research, particularly for studying liver function, metabolism, and toxicity). The results observed indicated that the antihaemolytic activities of these carotenoid extracts against H_2_O_2_-induced haemolysis in mouse erythrocytes were 3.9–6.3 times higher than β-carotene. In addition, a dose-dependent in vitro antiproliferative activity against HepG2 cells was reported for *H. limi* extracts, while that from *H. vescus* exhibited a relatively high activity in a dose-independent manner [42].

In other studies, carotenoids at a final concentration between 0.2 and 1.5 μM from a haloarchaeal strain (M8) reduced up to 50% hepatoma cell line (HepG2) viability in a concentration-dependent way. In addition, hepatoma cells exposed to haloarchaeal carotenoids were less sensitive to oxidative stress mediated by H_2_O_2_, which demonstrates the protective effect of the natural pigments [47]. The antiproliferative effect on hepatoma cells, as well as antihaemolytic activities against H_2_O_2_-induced haemolysis in mouse erythrocytes, were also reported for extracts obtained from *Halogeometricum limi* and *Haloplanus vescus* [42].

The antitumoral effect of *Natrialba* sp. M6 carotenoid extract was described for hepatoma cells (HepG2), Caco-2 (colon cancer), MCF-7 (breast cancer), and HeLa (cervical cancer) [48]. In the case of MCF-7 commercial cell lines, the expression of genes specific for apoptosis was analysed by the real-time PCR technique using cells exposed or not to BR-rich carotenoid extract. Both early and late apoptosis were increased significantly by about 10% and 39%, respectively, due to the upregulation of CASP3, CASP8, and BAX gene expression (all involved in the process of apoptosis or programmed cell death) in the MCF-7 cell line. In contrast, the expression of the genes MKI67 and SOX2 was significantly downregulated in the treated MCF-7 cell line (MKI67 is a marker for cell proliferation and a regulator of global gene expression and chromatin organisation, whilst the SOX2 gene is a transcription factor crucial for embryonic development and stem cell maintenance).

The antiproliferative effect on breast cancer cell lines has been explored in other studies [15]. In the case of carotenoid extracts from *H. mediterranei*, cell adhesion, viability, diameter, and cell concentrations were substantially reduced in cell lines representative of the four well-defined subtypes of breast cancer (Luminal A, Luminal B, HER2-enriched, and triple-negative) [49,50]. Similar studies were conducted with BR extracts from the haloarchaeon *Haloarcula* sp. A15 corroborated early and late apoptosis of breast cancer cells (MCF-7 cell line), probably connected to upregulation of CASP3, CASP8, and BAX gene expression and the downregulation of expression for the genes MKI67 and SOX2 [51].

More recently, the effect of BR-rich extracts from *H. mediterranei* on myeloid leukaemia (ML) cell lines, including chronic myelogenous leukaemia (CML) and acute myelogenous leukaemia (AML) models, has been monitored. BR-rich extracts showed selective cytotoxicity on tumoral cell lines, with minimal effects on normal peripheral blood mononuclear cells (PBMCs) from healthy donors, suggesting their potential for tumour-specific targeting. The morphological features of the cells treated with BR-rich extracts were monitored, observing significant changes such as the formation of apoptotic bodies, cell blebbing, and clumping, which evidenced the induction of apoptosis [52].

In summary, the described antiproliferative effects of BR-rich extracts from various haloarchaeal strains, notably on cell lines of hepatic and breast cancer, or leukaemia, suggest their potential as a valuable candidate for novel anticancer therapies. However, further investigations are required to translate these findings into biomedicine, the formulation of drugs, and clinical interventions.

### 4.3. Immunomodulatory/Anti-Inflammatory Activities of BR and BR-Rich Extracts

Although the effect of carotenoids in the human immune system has been extensively explored, the potential effects of BR as an immunomodulatory or anti-inflammatory compound are far from known. However, recent studies have described the anti-inflammatory activities and immunomodulatory benefits of BR on human commercial cell lines, showing promising results.

For example, a carotenoid-rich extract in BR and C18 fatty acids obtained from *Haloarcula* sp. isolated from Odiel Saltworks (south of Spain) showed potent antioxidant capacity using the ABTS assay. This study confirmed that the pretreatment with this carotenoid-rich extract of lipopolysaccharide (LPS)-stimulated macrophages caused a significant reduction in ROS production, a decrease in the pro-inflammatory cytokines TNF-α and IL-6 levels, and an upregulation of the factor Nrf2 and its target gene heme oxygenase-1 (HO-1). Nrf2 is a transcription factor that acts as a master regulator of the cellular antioxidant and detoxification response. It works by binding to specific DNA sequences called antioxidant response elements (AREs) to activate the expression of dozens of genes involved in protecting cells from oxidative stress, inflammation, and chemical damage. Consequently, these results suggest the use of this carotenoid extract as a therapeutic agent in oxidative-stress-related inflammatory disease treatments [43,45].

Similarly, a study carried out with BR from *Halorubrum tebenquichense* suggested that the combination of carotenoid with dexamethasone (Dex) in ultra-small macrophage-targeted nanoparticles could act as a potential intestinal repairing agent [53]. The ultra-small structures containing BR and Dex were extensively captured by macrophages and Caco-2 cells (a human epithelial cell line derived from colon adenocarcinoma that is widely used in research as a model for the intestinal barrier). The results confirmed high anti-inflammatory and antioxidant activities on Caco-2 cells and lipopolysaccharide-stimulated THP-1-derived macrophages, reducing 65% and 55% of TNF-α and IL-8 release, respectively, and 60% of ROS production. The ultra-small structures also reversed the morphological changes induced by inflammation and increased the transepithelial electrical resistance, partly reconstituting the barrier function. Thus, this work evidenced that the BR-Dex-containing nanostructure deserves further exploration as an intestinal-barrier-repairing agent [53]. More comments on immobilisations of BR on nanomaterials as an efficient approach to improve its delivery in the context of therapeutic treatments are described in Section 4.

Overall, while the literature on BR suggests the therapeutic potential of this carotenoid in mitigating oxidative-stress-related inflammatory diseases and promoting intestinal repair, it is important to note that the current number of studies reported is still limited, and therefore, more efforts are required in this field to establish robust conclusions.

### 4.4. Effects of BR and BR-Rich Extracts on Key Enzymes and Proteins Involved in Human Pathologies

Considering the role that BR plays in microbial strain producers, and taking into account the positive results observed when BR is used as an antitumoral or anti-inflammatory compound, several recently published studies have focused on exploring the effect of this carotenoid on enzymes and proteins that are key targets for the development of certain diseases in humans and, ultimately, for diagnosis/treatment.

At the beginning of this decade, some studies with BR from haloarchaeal strains isolated from saline lakes of the Atacama Desert proved that this carotenoid inhibited the enzymatic activity of cholinesterase (a key enzyme for diagnosis and monitoring of hepatic diseases, exposure to pesticides, etc.). The enzymatic assays were conducted using acetylcholinesterase (AChE) and butyrylcholinesterase (BuChE), identifying an AChE inhibition IC_50_ of 2.96 ± 0.08 μg/mL and BuChE inhibition IC_50_ of 2.39 ± 0.09 μg/mL for the most active BR extract (from *Halorubrum tebenquichense* Te Se-85). These assays were complemented with docking calculations, from which it was possible to conclude that BR can exert their inhibitory activity, fitting into the enzyme pocket by their halves, in the presence of cholinesterase dimers [54]. *Halobacterium salinarum* and *Haloarcula hispanica* carotenoid extracts can also inhibit acetylcholinesterase apart from the enzyme tyrosinase, and, therefore, they could have potential applications as a treatment for inflammatory, neurological, and dermatological diseases [37].

By using BR-rich extracts from *H. mediterranei* cells grown under different nutritional conditions, the effect on key enzymes such as α-glucosidase, α-amylase, and lipase enzymes was also assessed to determine if they could be used to reduce blood glucose and lipid absorption. The highest inhibitory activity has been detected in extracts obtained from haloarchaeal cells grown in the presence of 2.5% glucose or starch cell cultures, with IC_50_ values of 5.3 µg/mL, which was considerably lower than those observed in the internal control (78.4 µg/mL) and those from the commercial drug Orlistat (37.5 µg/mL), respectively. Lipase inhibition by *H. mediterranei* carotenoid extracts was stronger than by others previously reported. One of the key messages of this work was that the composition of the BR-rich extracts is different when comparing extracts isolated from *H. mediterranei* grown under different C/N ratios [34,35].

A recent work conducted with BR-rich extracts isolated from *Arthrobacter agilis* (a bacterium isolated from snowflakes) demonstrated that the incorporation of the extracts in a cream to provide skin protection against UV exposure resulted in protection of the enzyme alkaline phosphatase against oxidative and heat stresses, increasing the temperature at which the bovine serum albumin (BSA) is denatured. In addition, the use of this BR-rich extract also partially prevented elastin aggregation induced by salt stress. A deep analysis of this process revealed that the use of BR-rich cream by 23 smokers for 28 days reduced protein carbonylation (a marker of oxidative stress in the proteome) and elastin degradation in human primary keratinocytes and in ex vivo skin explants, thanks to a dual mode of action: BR as an antioxidant and as a chaperon-like protein [55]. Similarly, carotenoids from *Halorhabdus utahensis* reached 90% hyaluronidase inhibition with 1.5 μg, confirming relevant potential for applications in the skin care sector [31].

## 5. Formulations for the Delivery of Bacterioruberin as Part of Therapeutic Applications

As the studies on the potential uses of BR in biomedicine progress promisingly, a new innovative research question arises as a challenge in connection to BR-based formulations and their delivery to support therapeutic applications [56,57,58]. In this context, the use of nanomaterials (mainly nanovesicles, micelles, or liposomes, but also green metallic nanoparticles) is revealed as the best strategy to mobilise and deliver BR in human cells and tissues [58,59]. A recent study has developed an inhalable polymeric-lipid nanocapsule formulation with mucus-penetrating properties to co-encapsulate Pirfenidone (Pfd) and BR, aiming to enhance anti-inflammatory, antioxidant, and antifibrotic effects by modulating pulmonary macrophage activity. The nanocapsules with immobilised Pfd and BR (pNC-BR-Pfd) preserved their physicochemical properties after nebulisation using a vibrating mesh nebuliser. The results demonstrated that pNC-BR-Pfd capsules reduced intracellular reactive oxygen species and suppressed IL-6 and TNF-α levels by approximately 3-fold at 10 μg/mL Pfd and 0.64 μg/mL BR, whilst free Pfd only partially inhibited IL-6 and did not affect TNF-α [60].

Other studies have successfully achieved the synthesis of nanovesicles embedding BR, demonstrating that the biological activity and stability of BR improve substantially [53,59]. Regarding these formulations, a type of nanovesicles termed TA-nanoarchaeosomes (which are made of polar archaeolipids) and Tween 80 (TA: polar archaeolipids (PAs): Tween 80, 5:5:4 *w*:*w*:*w*, TA-nanoARC) was used as carriers of BR to explore the effect of those complexes on commercial cells that are representative of human lung epithelial cells (A549 cell line) and human monocytic cells (THP-1 cell line). The results demonstrated that the nanovesicles were stable to storage and nebulization. In addition, TA-nanoARC showed cytotoxic effect on A549 cells after 48 h, with an IC_50_ of 0.15 μg/mL (~0.20 µM). Such cytotoxicity was exerted at a concentration harmless to mTHP-1 cells. In addition, the conditioned medium from TA-nanoARC nebulised on A549 cells reduced the expression of the CD204/SRA-1, an M2 phenotype marker, and induced pro-inflammatory activity, comparable or even greater than that induced by lipopolysaccharide, including IL-6 and TNF-α, in mTHP-1 as a model of tumour-associated macrophages. The main conclusion from this work pointed out the internalisation of the highly viscous and ordered TA-nanoARC rich in neutral archaeolipids and subsequent lysosomal dysfunction (and not its antioxidant activity), as responsible for the negative impact on A549 cells [61]. These nanostructures based on archaeolipids as carriers have also been used for topical administration of vitamin D3 and BR in the treatment of psoriasis. The results demonstrated anti-inflammatory and antioxidant activity of the nanostructures (which were stable over time and showed good occlusion capacity and spreadability) on bi-cellular spheroids, consisting of a fibroblast core surrounded by a ring of keratinocytes activated with imiquimod (IMQ) as a psoriasis model. The nanostructures did not decrease the viability of spheroids but significantly reduced the release of pro-inflammatory cytokines (IL-8, IL-6, and TNF-α), ROS, and matrix metalloproteinases from IMQ-induced spheroids to levels similar to the uninduced spheroids [62]. In a more recent work, the same authors have improved these nanostructures (macrophage-targeted nanostructured archaeolipid carriers made of a compritol, C_50_ dipolar carotenoid bacterioruberin (BR), and vitamin D3 (VD3) core, covered by sn 2,3 ether-linked polar archaeolipids extracted from the haloarchaeon *Halorubrum tebenquichense* and Tween 80) in an effort to reduce the use of topical corticosteroids in psoriasis and atopic dermatitis. Results obtained clearly justify the potential use of these nanocomplexes as a promising topical anti-inflammatory agent with wound healing and antimicrobial activities that deserve future in vivo exploration [63].

Metal oxide nanoparticles (NPs) have also been described as nanomaterials useful to carry carotenoids, including BR. In 2015, the immobilisation of BR on nanomaterials (together with the immobilisation of the protein bacteriorhodopsin) was described for the first time. On this occasion, these two biomolecules were used as sensitisers by immobilising them on nanoporous titanium dioxide films successfully and employing them as molecular sensitisers in dye-sensitised solar cells with efficient photocurrent generation as an alternative to silicon crystalline solar cells [64]. A few years later, this research line reported new results on the optimisation of green synthesis of TiO_2_ NPs in combination with the immobilisation of BR and other carotenoids to improve photoelectric conversion efficiently. The results revealed the highest photoelectric conversion efficiency (η) of 0.44%, which was almost as good as natural dye-sensitised solar cells [22]. Insofar as these nanoparticles with immobilised BR have proven stable and with solar sensitised activity, it would be feasible to think of NPs as carriers of BR so that their administration (perhaps by topical injection and/or dermal applications) could be more efficiently localised and controlled.

## 6. Potential Food-Related Applications of Bacterioruberin

Most of the documents that reported on BR refer to its chemical features, its biological roles in microorganisms, or potential applications of BR in biotechnology and biomedicine. However, in connection with the application, there is a notable lack of scientific investigation regarding its applicability in the food industry, a sector in which this carotenoid could be of interest, not only due to its intense pigment colour, but also due to its high antioxidant capacity. Thanks to its pigmenting capacity, BR could enhance the aesthetic appeal of food products, such as surimi seafood, food colouring for baking, candy, etc. To address and make these uses successful, more efforts must be made to gather enough data regarding their stability, compatibility with diverse food matrices, and potential biochemical interactions. In addition, the analysis of the behaviour of this carotenoid under different processing conditions, its potential impact on sensory attributes, and its overall safety profile is pivotal for its industrial use.

So far, only a few papers and a publicly available doctoral thesis have explored the use of BR as a colourant in surimi sticks [65]. Artificial colourants are usually added to surimi seafood to mimic shellfish or salmon colour. When exposed to heating and light, the stability of BR from the haloarchaeon *Halobacterium salinarum* HM3 was evaluated in soybean oil and added to surimi from bigeye snapper (*Priacanthus tayenus*). In addition, the acute oral toxicity was monitored in rats. The results revealed that the exposure to high temperatures (70–90 °C) led to a decrease in BR content (~15–25%) and scavenging activity (~30%) after 24 h. Regarding light exposure, around 10% of BR was degraded after exposure at 5000 lux for 24 h. In terms of acute oral toxicity, female rats showed a decrease in body weight gain when treated with the highest concentration tested (1000 mg/kg/day), but no deaths or other adverse effects occurred after 14 days of BR treatment. BR was also tested as a colour additive in surimi gels by adding 200 mg/kg. Under these conditions, the final product displayed an orange-pink colour with the L*, a*, and b* values in the 67.6, 31.7, and 23.5 ranges, respectively. These values coincide with surimi gels dyed with cantaxanthin (250 mg/kg, 50.2, 35.7, and 24.9) and paprika oleoresin (51.6, 30.3, and 26.2). Furthermore, after 7 days of refrigerated storage under lighting, no colour changes were observed in surimi gels stored in aluminium foil. In contrast, colour differences were detected after 5 days when a polypropylene bag was used for storage. Finally, BR could prevent lipid peroxidation in surimi gels during storage [65]. On the other hand, haloarchaeal BR has been used to improve colour and reduce fat oxidation in pangasius emulsion sausage during refrigerated storage. The BR was incorporated as 10 mg/kg (T1), 25 mg/kg (T2), and 50 mg/kg (T3) in the emulsion sausages, and the main results obtained revealed that BR application made possible the storage under optimal conditions till the 42nd, preventing the oxidation of lipids without affecting the gel strength of emulsion sausages [66].

Globally, there is a growing number of consumers seeking natural products and ingredients produced sustainably. It is in this context that BR emerges as a compound, expanding the range of ingredients that could be incorporated into processed foods and nutraceuticals [67]. Apart from being an interesting natural colourant, BR could be used as an additive for functional food due to its strong antioxidant activity, which will contribute to the replacement of pigments of chemical synthesis as a response to the growing demand for healthy food and drinks (including chocolate candies, fast-food strawberry shakes, and several ready-to-eat cereal brands, baby foods, snacks, and beverages). This is an entirely new field of study, and at the time of writing, there is no evidence of studies describing the incorporation of BR into nutraceuticals or functional foods.

More recently, other uses of BR have been described in connection with post-harvest losses and rapid fruit ripening at room temperature, which are major challenges in preserving fruit quality. In this context, BR (2%) from *Arthrobacter* sp. isolated from Xinjiang desert was applied to fresh peaches, grapes, and apricots. This application effectively prevented spoilage for six days at room temperature [68].

## 7. Conclusions

BR is a natural pigment described decades ago, whose benefits and potential uses have become evident mainly during the last decade, thus emerging as a promising biomolecule that could have a wide range of applications across the fields of nutraceuticals, biomedicine, food processing and conservation, pharmaceuticals, and textiles [24,69,70]. Its natural origin from microorganisms aligns with the growing consumer demand for eco-conscious and sustainable products.

**Figure 2:**
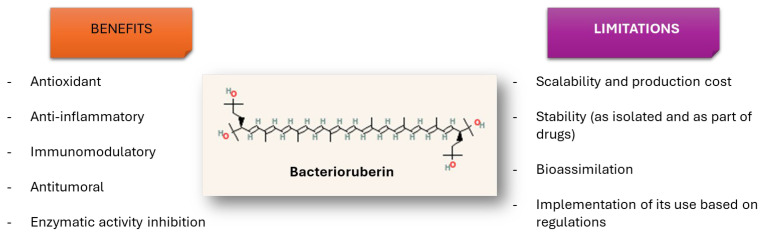
Main current benefits and limitations of the use of bacterioruberin in the medical sector.

Due to the antioxidant, anti-inflammatory, immunomodulatory, and antitumoral activities of BR, this C_50_ carotenoid offers new approaches and strategies for developing new drug formulations or drug immobilisation techniques as part of the treatment of human pathologies related to the immune system and cancer, among others (Figure 2). A better promotion of more research on haloarchaeal pigments would make it possible to uncover novel applications for these promising C_50_ carotenoids and to overcome some of the major limitations that the use of BR still has in medical practice. Within the main challenges to be addressed shortly, the following can be highlighted:

- It is necessary to accurately identify the percentage that the BR represents in all the described carotenoid cell extracts to determine to what extent the observed biological activities depend on said carotenoid or on its closely related carotenoids, bisanhydrobacterioruberin (BABR), and monoanhydrobacterioruberin (MABR). This is one of the challenges that should be addressed in the short term.
- It is recommended to improve the funding for basic research to elucidate the underlying molecular mechanisms that explain the effects of BR on human cells and tissues, which would guarantee faster progress in the biomedical application of this natural pigment. As the molecular mechanisms of BR become better understood regarding its interaction with different human cell and tissue types, more personalised and effective formulations could be developed.
- It is important to emphasise those studies that are optimising the synthesis of nanomaterials that could act as carriers for BR to improve the release of this natural compound in the human body, thus enhancing its impact on the treated area. Some haloarchaea species can synthesise green nanoparticles (NPs) and BR; therefore, approaches that combine both compounds obtained from the same species to develop an efficient and sustainable delivery strategy would even foster green chemistry and circular economy processes that would benefit the biomedicine and biotechnology industries.
- It is essential to continue with studies that allow for the (i) assessing the safety and efficacy of BR in human clinical settings (all this in alignment with health regulations; it is relevant to address BR bioavailability, metabolic reactions involved on its assimilation, or pharmacokinetics in humans or animals, thus making it possible to accurately assess potential toxicity, safety profile, or maximum tolerated doses); (ii) enabling a better transfer of knowledge between research laboratories and the sustainable pharmaceutical industry or industries related to nutraceuticals or processed food to integrate BR into their formulations; and (iii) ensuring compliance with the regulations that apply to the use of these natural compounds in therapeutic strategies.
- It is relevant to make efforts to ensure the incorporation of BR into foods in a way that could guarantee its stability in real food matrices, and oversee its sensory impact and its relevance for human nutrition. In addition, it will be important to monitor its bioavailability, absorption, metabolism, and utilisation in human pathways of BR, comparing it with classical dietary carotenoids.
- It could be interesting to explore other potential uses of BR in biomedicine apart from those described, depending on the biological activities already reported for BR. As an example of a recent new potential application, it has been reported that BR and selenium nanoparticles produced by *Haloferax alexandrinus* GUSF-1 (KF796625) show antimicrobial activity and ameliorate arsenic toxicity in human lymphocytes [67].
- Finally, the feasibility of incorporating bacterioruberin into food matrices or supplements, including its stability under processing conditions and regulatory status, should be further explored.

## Figures and Tables

**Table 1:** Structures, and common and scientific names of bacterioruberin (BR), monoanhydrobacterioruberin (MABR), and bisanhydrobacterioruberin (BABR) (adapted from [19,22]).

Common Name Chemical Formula	Chemical Structure (Stereoisomers)
Bacterioruberin C_50_H_76_O_4_	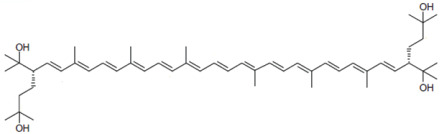 (2*S*,2′*S*)-2,2′-bis(3-hydroxy-3-methylbutyl)-3,4,3′,4′-tetradehydro-1,2,1′,2′-tetrahydro-γ,γ-carotene-1,1′-diol
Monoanhydrobacterioruberin C_50_H_74_O_3_	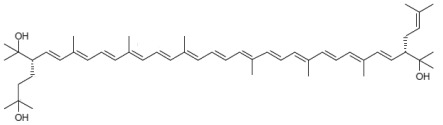 (3*S*,4*E*,6*E*,8*E*,10*E*,12*E*,14*E*,16*E*,18*E*,20*E*,22*E*,24*E*,26*E*,28*E*,30*S*)-30-(2-hydroxypropan-2-yl)-2,6,10,14,19,23,27,33-octamethyl-3-(3-methylbut-2-en-1-yl)tetratriaconta-4,6,8,10,12,14,16,18,20,22,24,26,28-tridecaene-2,33-diol
Bisanhydrobacterioruberin C_50_H_72_O_2_	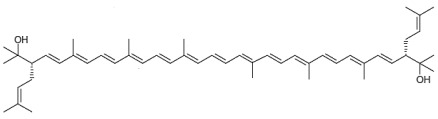 (3*S*,4*E*,6*E*,8*E*,10*E*,12*E*,14*E*,16*E*,18*E*,20*E*,22*E*,24*E*,26*E*,28*E*,30*S*)-2,6,10,14,19,23,27,31-octamethyl-3,30-bis(3-methylbut-2-en-1-yl)dotriaconta-4,6,8,10,12,14,16,18,20,22,24,26,28-tridecaene-2,31-diol

## Data Availability

No new data were created or analyzed in this study. Data sharing is not applicable to this article.
